# Supplementary material for: Multi-channel recordings reveal age-related differences in the sleep of juvenile and adult zebra finches
Source: Sci Rep. 2023 May 27;13:8607. doi: 10.1038/s41598-023-35160-1 (PMC10224955; doi:10.1038/s41598-023-35160-1)
Supplement: Supplementary file 2 — Supplementary Information 2. [file 41598_2023_35160_MOESM2_ESM.pdf]

Multi-channel recordings reveal age-related differences in the sleep of juvenile and adult zebra finches

Hamed Yeganegi[1,2] and Janie M. Ondracek[1*]

[1]Chair of Zoology, Technical University of Munich, Liesel-Beckmann-Str. 4, 85354 Freising-Weihenstephan, Germany

[2]Graduate School of Systemic Neurosciences, Ludwig-Maximilians-University Munich, Großhaderner Str. 2, 82152 Planegg, Germany

Analysis of Variance tables for the tests presented in the main text

Two-way ANOVA for **(δ+θ)**/γ, x1: age, x2: sex, using anovan MATLAB function

Source	Sum Sq.	d.f.	Mean Sq.	F	Prob>F
X1	4138.6488	1	4138.6488	6.2945	0.032698
X2	141.6964	1	141.6964	0.21551	0.64568
Error	30245.2729	46	657.5059		
Total	34977.0134	48			

Constrained (Type III) sums of squares.

Two-way ANOVA for **(δ+θ)**, x1: age, x2: sex, using anovan MATLAB function

Source	Sum Sq.	d.f.	Mean Sq.	F	Prob>F
X1	0.015669	1	0.015669	3.2951	0.076009
X2	0.0084312	1	0.0084312	1.773	0.18957
Error	0.21874	46	0.0047553		
Total	0.23547	48			

Constrained (Type III) sums of squares.

Two-way ANOVA for γ, x1: age, x2: sex, using anovan MATLAB function

Source	Sum Sq.	d.f.	Mean Sq.	F	Prob>F
X1	1.3207e-05	1	1.3207e-05	0.51222	0.4778
X2	3.5236e-05	1	3.5236e-05	1.3665	0.24843
Error	0.0011861	46	2.5784e-05		
Total	0.0012218	48			

Constrained (Type III) sums of squares.

Two-way ANOVA for **REM percentage**, x1: age, x2: sex, using anovan MATLAB function

Source	Sum Sq.	d.f.	Mean Sq.	F	Prob>F
X1	620.137	1	620.137	10.7135	0.0027508
X2	372.7795	1	372.7795	6.4402	0.01679
Error	1678.6209	29	57.8835		
Total	2380.5423	31			

Constrained (Type III) sums of squares.

Two-way ANOVA for **SWS percentage**, x1: age, x2: sex, using anovan MATLAB function

Source	Sum Sq.	d.f.	Mean Sq.	F	Prob>F
X1	308.5916	1	308.5916	5.4826	0.026288
X2	342.2265	1	342.2265	6.0801	0.019828
Error	1632.2957	29	56.2861		
Total	2081.2443	31			

Constrained (Type III) sums of squares.

Two-way ANOVA for **IS percentage**, x1: age, x2: sex, using anovan MATLAB function

Source	Sum Sq.	d.f.	Mean Sq.	F	Prob>F
X1	2243.3562	1	2243.3562	28.6299	9.573e-06
X2	1344.5007	1	1344.5007	17.1586	0.0002715
Error	2272.3579	29	78.3572		
Total	4809.4547	31			

Constrained (Type III) sums of squares.

Two-way ANOVA for **REM duration**, x1: age, x2: sex, using anovan MATLAB function

Source	Sum Sq.	d.f.	Mean Sq.	F	Prob>F
X1	15.4459	1	15.4459	6.3213	0.017732
X2	13.1744	1	13.1744	5.3917	0.027459
Error	70.8609	29	2.4435		
Total	90.6333	31			

Constrained (Type III) sums of squares.

Two-way ANOVA for **SWS duration**, x1: age, x2: sex, using anovan MATLAB function

SourceSum	Sq.	d.f.	Mean Sq.	F	Prob>F
X1	14.0336	1	14.0336	11.7447	0.0018455
X2	14.2408	1	14.2408	11.918	0.0017279
Error	34.6518	29	1.1949		
Total	54.1352	31			

Constrained (Type III) sums of squares.

Two-way ANOVA for **IS duration**, x1: age, x2: sex, using anovan MATLAB function

SourceSum	Sq.	d.f.	Mean Sq.	F	Prob>F
X1	14.4968	1	14.4968	6.7135	0.014823
X2	11.7487	1	11.7487	5.4409	0.026817
Error	62.6208	29	2.1593		
Total	80.7874	31			

Constrained (Type III) sums of squares.

One-way ANOVA for **REM Percentage**, x1: juvenile sex, using anovan MATLAB function

Source	Sum Sq.	d.f.	Mean Sq.	F	Prob>F
X1	372.78	1	372.78	4.42	0.0529
Error	1265.7	15	84.38		
Total	1638.48	16			

One-way ANOVA for **SWS Percentage**, x1: juvenile sex, using anovan MATLAB function

Source	Sum Sq.	d.f.	Mean Sq.	F	Prob>F
X1	342.23	1	342.227	3.91	0.0667
Error	1312.77	15	87.518		
Total	1655	16			

One-way ANOVA for **IS Percentage**, x1: juvenile sex, using anovan MATLAB function

Source	Sum Sq.	d.f.	Mean Sq.	F	Prob>F
X1	1344.5	1	1344.5	9.21	0.0084
Error	2189.49	15	145.97		
Total	3533.99	16			

One-way ANOVA for **REM Duration**, x1: juvenile sex, using anovan MATLAB function

Source	Sum Sq.	d.f.	Mean Sq.	F	Prob>F
X1	21.3391	1	21.3391	54.31	2.33001e-06
Error	5.8933	15	0.3929		
Total	27.2324	16			

One-way ANOVA for **SWS Duration**, x1: juvenile sex, using anovan MATLAB function

Source	Sum Sq.	d.f.	Mean Sq.	F	Prob>F
X1	28.5115	1	28.5115	117.58	1.70414e-08
Error	3.6371	15	0.2425		
Total	32.1486	16			

One-way ANOVA for **IS Duration**, x1: juvenile sex, using anovan MATLAB function

Source	Sum Sq.	d.f.	Mean Sq.	F	Prob>F
X1	12.4023	1	12.4023	34.76	2.93802e-05
Error	5.3517	15	0.3568		
Total	17.754	16			

Two-way ANOVA for **local wave occurrence rate**, x1: age, x2: sex, using anovan MATLAB function

Source	Sum Sq.	d.f.	Mean Sq.	F	Prob>F
X1	0.78796	1	0.78796	25.1373	6.7767e-07
X2	0.19424	1	0.19424	6.1967	0.013031
Error	21.8798	698	0.031346		
Total	23.6767	700			

Constrained (Type III) sums of squares.

Two-way ANOVA for **connectivity across sleep stages in adults**, x1: [SWS, IS, REM stages], x2: [LL, RR, LR connectivity], using anova2 MATLAB function

Source	SS	df	MS	F	Prob>F
Columns	7.0207	2	3.5103	239.7073	1.4414e-52
Rows	0.075048	2	0.037524	2.5624	0.079799
Interaction	0.054864	4	0.013716	0.93661	0.44387
Error	2.7678	189	0.014644		
Total	9.9184	197			

Two-way ANOVA for **connectivity across sleep stages in juveniles**, x1: [SWS, IS, REM stages], x2: [LL, RR, LR connectivity], using anova2 MATLAB function

Source	SS	df	MS	F	Prob>F
Columns	0.021983	2	0.010991	0.53371	0.5874
Rows	2.8498	2	1.4249	69.1902	9.6389e-23
Interaction	0.0043447	4	0.0010862	0.052742	0.99476
Error	3.5216	171	0.020594		
Total	6.3978	179			

Two-way ANOVA for **REM-to-IS** transitions, x1: age, x2: sex, using anovan MATLAB function

Source	Sum Sq.	d.f.	Mean Sq.	F	Prob>F
X1	2326266.7508	1	2326266.7508	27.0961	1.3043e-05
X2	1315662.4251	1	1315662.4251	15.3247	0.00048249
Error	2575573.0455	30	85852.4348		
Total	5157324.2424	32			

Two-way ANOVA for **REM-to-SWS** transitions, x1: age, x2: sex, using anovan MATLAB function

Source	Sum Sq.	d.f.	Mean Sq.	F	Prob>F
X1	390367.1928	1	390367.1928	4.8759	0.03502
X2	454779.787	1	454779.787	5.6804	0.023687
Error	2401833.4924	30	80061.1164		
Total	2983183.5152	32			

Two-way ANOVA for **SWS-to-IS** transitions, x1: age, x2: sex, using anovan MATLAB function

Source	Sum Sq.	d.f.	Mean Sq.	F	Prob>F
X1	1556397.3064	1	1556397.3064	46.906	1.3373e-07
X2	623673.0918	1	623673.0918	18.796	0.0001508
Error	995435.3788	30	33181.1793		
Total	2612580.2424	32			

Two-way ANOVA for **SWS-to-REM** transitions, x1: age, x2: sex, using anovan MATLAB function

Source	Sum Sq.	d.f.	Mean Sq.	F	Prob>F
X1	341905.4848	1	341905.4848	4.8873	0.034822
X2	347332.5143	1	347332.5143	4.9649	0.033512
Error	2098746.0152	30	69958.2005		
Total	2571704.2424	32			

One-way ANOVA for **SWS-to-REM** transitions, x1: juvenile sex, using anovan MATLAB function

Source	Sum Sq.	d.f.	Mean Sq.	F	Prob>F
X1	2412.03	1	2412.03	8.41	0.011
Error	4299.78	15	286.65		
Total	6711.82	16			

One-way ANOVA for **REM-to-SWS** transitions, x1: juvenile sex, using anovan MATLAB function

Source	Sum Sq.	d.f.	Mean Sq.	F	Prob>F
X1	3158.19	1	3158.19	7.42	0.0157
Error	6380.4	15	425.36		
Total	9538.59	16			

One-way ANOVA for **REM-to-IS** transitions, x1: juvenile sex, using anovan MATLAB function

Source	Sum Sq.	d.f.	Mean Sq.	F	Prob>F
X1	9136.5	1	9136.54	9.82	0.0068
Error	13956.2	15	930.41		
Total	23092.7	16			

One-way ANOVA for **SWS-to-IS** transitions, x1: juvenile sex, using anovan MATLAB function

Source	Sum Sq.	d.f.	Mean Sq.	F	Prob>F
X1	4331.06	1	4331.06	20.14	0.0004
Error	3226.43	15	215.1		
Total	7557.49	16			

Two-way ANOVA for **CC** in **LL, RR,** and **LR** pair of channels, in **adults**, x1: SWS, IS, REM, x2: LL, RR, and LR, using anova2 MATLAB function

Source	SS	df	MS	F	Prob>F
Columns	7.0207	2	3.5103	239.7073	1.4414e-52
Rows	0.075048	2	0.037524	2.5624	0.079799
Interaction	0.054864	4	0.013716	0.93661	0.44387
Error	2.7678	189	0.014644		
Total	9.9184	197			

Accompanying pairwise comparisons from Tukey multiple comparison test (multcompare function):

Group1	Group2	p-value
LL	RR	0.0396

LL	LR	9.56e-10
RR	LR	9.56e-10

Two-way ANOVA for **CC** in **LL, RR,** and **LR** pair of channels, in **juveniles**, x1: SWS, IS, REM, x2: LL, RR, and LR, using anova2 MATLAB function

Source	SS	df	MS	F	Prob>F
Columns	2.8498	2	1.4249	69.1902	9.6389e-23
Rows	0.021983	2	0.010991	0.53371	0.5874
Interaction	0.0043447	4	0.0010862	0.052742	0.99476
Error	3.5216	171	0.020594		
Total	6.3978	179			

Accompanying pairwise comparisons from Tukey multiple comparison test (multcompare function):

Group1	Group2	p-value
LL	RR	0.5489
LL	LR	9.56e-10
RR	LR	9.56e-10
